# Liquorice Health Check, Oro-Dental Implications, and a Case Report

**DOI:** 10.1155/2009/170735

**Published:** 2009-07-08

**Authors:** Louis Z. G. Touyz

**Affiliations:** Department of Periodontics Faculty of Dentistry, McGill University, 3640 University Street, Montreal, QC, Canada H3Y 2B2

## Abstract

Liquorice has an active substance, Glycyrrhizin which inhibits the conversion of precursor cortisol to cortisone by inhibiting the enzyme *11-betahydroxysteroid dehydrogenase*. When imbibed, liquorice acts like hyperaldosteronism which presents with typical symptoms including high blood pressure, low blood potassium, and muscle pain and weakness. This article appraises physiological and pharmacological effects on health of liquorice, critiques products containing liquorice, describes a typical case report of liquorice-induced hypertension, and appraises oral effects from consumption of liquorice products.

## 1. Introduction

Liquorice, or liquorice, is a uniquely tasting herb derived from *Glycyrrhiza glabra*, and has been used in medicine for thousands of years. Liquorice is used as a flavorant in a variety of edibles, medicine, and tobacco, and is often innocently consumed in vast amounts without any regard or only with vague concepts of side effects liquorice may produce.

## 2. Aim

This paper appraises liquorice provides a reality check of its properties (botanical sources, chemical structure, active liquorice ingredient, physiological/pharmacological activity, some common liquorice containing consumables), their systemic impact on health, a typical case report of liquorice-induced hypertension, and effects of consumption of liquorice on oro-dental structures. Important clinical management principles for moderating liquorice consumption are suggested.

## 3. Liquorice

### 3.1. Botany

It is the roots (rhizomes) and stolons of *Glycyrrhiza glabra * (a.k.a Sweet Root, Spanish or Italian Liquorice), which is the commonest variety source of liquorice. *G.g lepidota * is American wild liquorice, while *G.g violacea * and *G.g glandulifera * are Persian/Turkish and Russian varieties, respectively. *Glycyrrhiza uralensis * (a.k.a. Manchurian liquorice) is the species favoured for traditional Chinese Eastern Medicines. Liquorice flavours are also found in the plants like Fennel *(Foeniculum vulgare)*, Anise seeds (from *Pimpinella anisum)*, and other plants [1]. 

### 3.2. Active Ingredient

The active chemical ingredients imparting the unique liquorice taste are glycyrrhizic acid and its glucoside, glycyrrhizin (C_42_H_62_O_16_). These molecules are regarded as nearly synonymous, are powerful organoleptic flavorants, and impart characteristic liquorice taste and aroma to mixtures in small concentrations [2].

### 3.3. Properties, Physiology and Pharmacology

Glycyrrhizin is 50 times sweeter than sucrose. It retains, when sapid, a singular liquorice flavour. The liquorice sweetness has a slower onset than sugar and lingers. Unlike artificial sweeteners like aspartame, saccharine, and cyclamates, it contains no sulfur molecule [3], and retains its sweetness when heated [2]. On hydrolysis glycyrrhizin yields 2 mols of glucuronic acid and 1 of glycyrrhetenic acid, a pentacyclic, tri-terpene which structure partially resembles that common to steroids, with a moiety attached.

Glycyrrhizin inhibits the conversion of the precursor cortisol to cortisone by inhibiting the enzyme *11-betahydroxysteroid dehydrogenase * [4]. Hydrolysis of slowly absorbing glycyrrhizin into the more rapidly absorbed glycyrrhetenic acid is performed by intestinal microbiota. Consequently antibiotics affecting gut flora, adversely affects absorption of liquorice. Liquorice boosts cellular formation of endogenous interferon, and has a positive long-term healing effect on Hepatitis-C-infected patients [5–9].

### 3.4. Posology and Dosage

Liquorice is marketed in various forms, and because its often sold as the natural grown product, concentrations in the plant varies. Solid extract 250–500 mg, three times daily, is suggested for medicinal purposes. Dried root is dispensed at 1–4 g, three times daily to a maximum of 12 g [2]. At 75 mg daily glycyrrhetenic acid (derived from 50 G/day liquorice), a raising effect on blood pressure is noted after 2 weeks. More than this, daily dose increases blood pressure proportional to increased liquorice intake [2, 4].

### 3.5. Liquorice Toxicity

This on its own is rare, yet not infrequent when encountered clinically and usually occurs in *diuretic medicated patients * unwittingly combining consumption of commercial products containing high amounts of liquorice extract like chewing tobacco, laxatives, or confections with concentrated liquorice extracts [9–11]. 

Consumption of glycyrrhizin is considered safe at 200 mg per day, a dose accepted as recommendation to Japanese. The accepted daily intake (ADI) for glycyrrhizin at 0.2 mg/kg/day is deemed safe; up to 1200 mg/day liquorice flavonoid oil shows no clinical noteworthy change of hematological or related biochemical parameters [12–14]. 

In the United States of America, glycyrrhizin is classified “as generally recognised as safe” as a flavouring agent, although not as a sweetener [2].

 Commercially liquorice flavoured sweets rarely have any serious medicinal side effects, especially if consumed irregularly, in moderate amounts, of less than 25 g of liquorice per day. 

## 4. Medical Impact

Liquorice has many positive and negative health modulating physiological effects which explain a variety of its medical effects. The most widely renowned negative action derives from liquorice's association with hypertension. Because of the inhibition of *11-beta hydroxysteroid dehydrogenase*, by liquorice, cortisol levels are high within the collecting duct of the kidney, and potassium is excreted while sodium is retained [4]. Cortisol has high mineralo-corticoid properties, that is, it acts like aldosterone and increases sodium re-absorption from the glomerular filtration in the proximal tubules of the kidney in ENaC channels [3, 4]. Sodium retention leads to higher osmotic intravascular pressure, which in turn retains more water, which increases circulating blood volume with consequent increased blood pressure leading to hypertension [4, 15].

Liquorice has a variety of positive healthy effects on the body. For example, it is known that Liquorice has antiviral properties and has some inhibitory effects on HIV, encephalitis, and SARS-corona viruses [11, 16, 17]. 

Also Glycyrrhetenic acid itself has a retardant effect on peptic ulcers, possibly due to the fact that it has antibacterial properties and retards the growth of *Helicobacter pylori * [18].

Other medicinal claims are that liquorice is anti-ulcer (peptic, duodenal, and aspirin) [19, 20]. Liquorice-induced hypokalemic myopathy may explain why GIT spasms relax, and also why liquorice containing alcohols are said not to induce emesis [21]. With gastric smooth muscle paralysis, the irritating effect of ethanol is reduced and gastric contraction is temporarily weakened. Liquorice among others is claimed to also be anti-inflammatory, an immune-stimulant, a demulcent, an expectorant, anticatarrhal, hepato-protective, a GIT spasmolytic, a mild laxative, and an antioxidant [20–24]. 

### 4.1. Consumption Products

**Figure 1:**
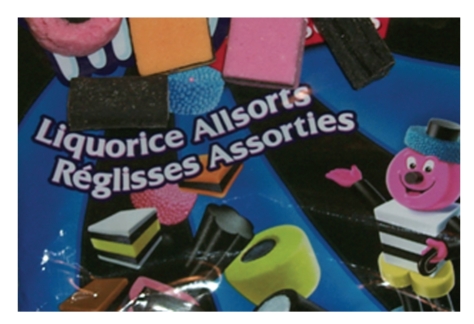
Ubiquitous Liquorice all sorts. A mainly carbohydrate and liquorice confection (by Maynards-Bassetts. Cadbury Adams, Toronto Ont.) These sweets are marketed freely to the public.

**Figure 2:**
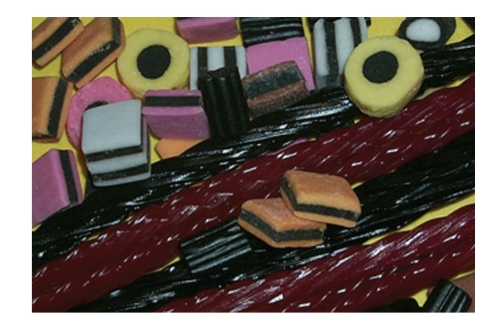
Liquorice All-Sorts with Red and Black Liquorice Twirls (by Allan Candy Toronto Ont.) and black Panda Liquorice Tubes (Elco Fine Foods, Richmond Hill, Ont.). These sweets are marketed freely to the public.

**Figure 3:**
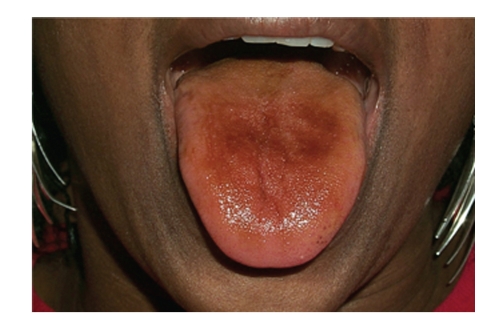
Short lived staining of the tongue after sucking black Liquorice confection. This stain is water soluble and usually disappears after a few hours.

Liquorice used as a flavorant in candies adds much gustatory joy to the variety of living pleasures. The flavour is so positive and pleasant, it is also used for flavouring other foods like ice-cream, biscuits, cakes, and drinks. It is a very popular flavour for sweet treats as “Liquorice All Sorts” (see Figures 1  and 2), and also in Salty Liquorice in Holland. Liquorice is added to baked confectionery, toffees, chocolates, chewing-gum, and sucking sweets. Black and Red Liquorice varieties are made using food dyes which can discolour the tongue. (Figure 3). Liquorice is also part of spice mixes constituting curry, and liquorice is also used in breath fresheners.

**Figure 4:**
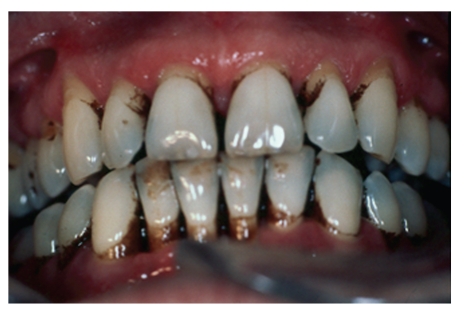
Heavy tobacco dental staining from pipe smoking with Liquorice as an additive. Gingival recession, alveolar bone loss, and periodontal pockets result from the deleterious effect of the tobacco smoke.

**Figure 5:**
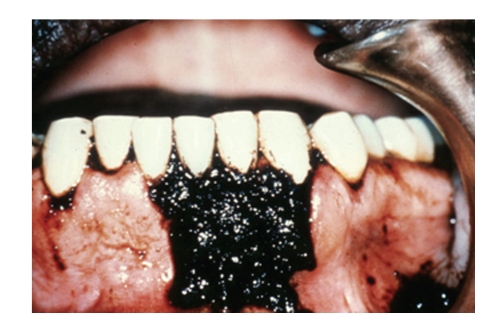
Ground Tobacco Wedge, flavored with Liquorice, is habitually placed into the labial Fornix adjacent to the lower incisors. This imparts a sense of euphoria deriving from the nicotine content of the tobacco, (not the liquorice), to its users.

**Figure 6:**
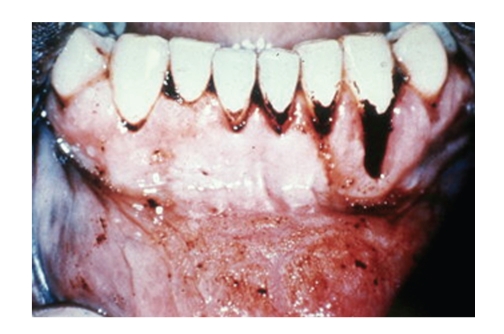
After removal of the Tobacco Wedge seen in Figure 5. There is cervical recession and staining, and keratotic changes to the adjacent mucosa. This is the result of prolonged contact of the Tobacco Wedge against the teeth and mucosa.

 It is also used in medications, in many syrups, lozenges, capsules, laxatives, cough-lozenges, and mixtures to mask bitterness and foul-flavours of other drugs. Liquorice is included in commercial over-the-counter tobacco products like pipe and chewing tobacco and snuffs. Liqourice can stain the tongue and teeth (see Figures 3, 4, 5  and 6).

Liquorice concentrate-flavour is popular in alcoholic drinks too like Absinthe (thujole containing) and Pernod in France, Anise in Europe, and Ouzo in Greece.

### 4.2. Liquorice Hypertension: A Case Report

A 55-year-old women presented with a brown/black tongue for routine dental check and maintenance. (See Figure 3). There was neither history of antibiotic use nor any current tobacco abuse. She had been diagnosed with hypertension two years previously. Despite multiple antihypertensive drugs, her blood pressure remained elevated. Preoperative dental of vital signs revealed blood pressure (BP) measures of Systolic 160 mm Hg/Diastolic 125 mm Hg. Although this was attributed to a possible “white-coat hypertension” phenomenon, she was referred to the hypertension clinic for further investigation and management of hypertension. Other than prescribed blood pressure-lowering drugs (diuretics, an ACE inhibitor and Beta-blocker), she was not taking any other medication or herbal products. She exercised regularly, denied excessive alcohol intake and consumed a “healthy” diet. On examination, casual sitting blood pressure was high, despite treatment. Routine laboratory investigations revealed hypokalemia. The diuretic was discontinued, K^+^ supplements were given and she was advised to consume a K^+^-rich diet. Two weeks after stopping the diuretic, plasma K^+^ was still low and hypertension was uncontrolled. The patient was investigated for hyperaldosteronism. Plasma aldosterone and rennin levels were very low. In light of hypokalemia and reduced plasma aldosterone levels, a diagnosis of pseudo-hyperaldosteronism was made. A detailed dietary history revealed that since the patient stopped smoking 4 years ago, she started eating liquorice regularly every day. This helped her relieve her cravings for tobacco. She always enjoyed eating liquorice, but since quitting smoking she consumed at least one pack of 200–250 g or more of black liquorice daily. The patient was advised to stop eating liquorice and to continue her K^+^-rich diet and K^+^ supplementation. Three months later K^+^ blood level was normal. Eighteen months after presentation at the hypertension clinic and after stopping liquorice consumption, her blood biochemistry remained normal and her blood pressure was controlled to within normal limits. Much lower doses of drugs than originally used kept her BP stable and she was clinically well. 

### 4.3. Oral and Dental Effects

Drug-induced local oral reactions are common [25]. A wide variety of drugs may induce one or more oral reactions including allergic reactions, aphthous-like lesions, burning mouth syndrome, glossitis, ulcerations, erythema multiforme, vesiculo-bullous lesions, color changes, oral lichenoid reactions, black hairy tongue, oral mucositis, gingival hyperplasia, salivary gland changes, dental changes, oral motor disorders, oral malodours, oral infections including osteonecrosis of the jaws, angioedema, and cheilitis [25]. Liquorice not only has local oral effects but also has systemic effects, as the above case report shows. 

Glycyrrhyzin by itself does not stain teeth, but when combined with dark food dyes, tobacco and/or curries, liquorice is associated with stains. Frequently liquorice is mixed with dark caramel and food colorings which leave a surface brownish/black tongue stain (Figure 3). It contributes to increased tobacco staining, especially when included in aromatic pipe tobaccos; the dental stain is directly proportional to the amount and frequency of the pipe smoking. Not only is the palatal and lingual side of teeth prone to accumulating dark tobacco stain but also the mucosa undergoes specific changes. (Figure 4) Combined with chewing tobacco, liquorice additives enhance and prolong the flavour of the chewing tobacco experience, and consequently damage from longer contact time onto the gingiva, seeming to derive more from tobacco contents rather than just liquorice. (Figures 5  and 6), Adjacent recession, cervical dentinal staining, and thickening with hyperkeratosis of mucosa are seen.

## 5. Discussion

Liquorice effects on the body are not widely acknowledged or understood by the public. This is because it is generally a pleasant experience and safe to eat liquorice flavoured candies, and although it is rare for someone to gormandise on them to reach toxic levels, as the case report indicates, this does occur.

Hypertension (as high blood pressure) is presumed to obtain when a measure of 130/90 mm mercury (Systolic/Diastolic pressure) or more is measured, normal being 120/70 mm mercury. Hypertension is among the major causes of morbidity and mortality in the world today, and because it is mainly symptomless and painless, hypertension has been labelled “the silent killer.” Atherosclerosis, myocardial infarction and stroke can all result from chronic undetected hypertension [15, 26–29].

A linear dose-related rise in blood pressure has been reported with liquorice consumption in various doses of (20–200 g a day for 4–2 weeks), corresponding to a daily intake of 75–540 mg glycerrhetinic acid [14]. This is an inordinate amount of daily liquorice consumption and would be regarded as a “Fad-diet” and not to be sustained for health.

 An acceptable daily intake of 0.015–0.229 mg glycyrrhizin/kg body weight/day has been proposed [30]. 

 Most problems derive from people on diuretic medications in combination with other sources of Liquorice. Hypokalemia partially paralyses smooth muscle contraction, and excess imbibing of liquorice-flavoured alcohols contributes to gastric paralysis, prevents emesis, and indirectly contributes to the development of alcoholism. Excess consumption affects blood pressure, kidney function, and gastrointestinal tract [30, 31]. Other than the proven aldosterone effect of liquorice, other medical ailments like headache, myalgia, and muscle fatigue also present [32, 33]. Liquorice contributes in part positively to all these, but eliminating liquorice may only improve, but not cure or totally prevent severe conditions presenting with these symptoms. Some herbal remedies and other common consumables like chewing gum containing liqourice may precipitate hypertension and associated hypokalemic symptoms [34, 35].

Liqourice-induced hypertension with hypokalemia must be differentiated from other genetic deficiencies which may present with similar findings: three monogenetic types of mineralo-corticoid hypertension have been identified, Liddle's syndrome, glucocorticoid-remediable hypertension, and apparent mineralo-corticoid excess, an autosomal recessive disorder with mutations in the 11*β*-HSD2 gene [36–39]. Use of chewing and sucking tobacco snuffs and other products containing liquorice-like herbal medications, teas, breath fresheners, chewing gums, alcoholic drinks, and food products, all contribute to chronic habitual frequent swallowing of large quantities of liquorice [34, 35].

Excess liquorice consumption contributes not only to deteriorating general health through potassium loss and sodium retention [40] but also to oro-dental compromise. Some people stop smoking and then help control tobacco cravings by consuming large quantities of liquorice as a chronic organoleptic stimulus to help quit. Tooth staining from black liquorice is known, but the tooth staining derives mainly from added dyes to liquorice confections and from liquorice-flavoured tobacco. Accumulation of extracellular polysaccharides from microbial activity contributes to biofilm formation and bacterial plaques. This allows for a tacky gummy surface of muco-polysaccharides to stick to stagnant areas on teeth, and with adherent chromogenic bacteria, liquorice tobacco products discolour teeth and accelerate adjacent gingival breakdown. Quitting the tobacco habit with safe stain removal through scaling and polishing from teeth is feasible.

Liquorice sweets are generally health promoting, pleasurable to eat, and in moderation on their own rarely stain teeth. Health care workers, including all in the dental team, discovering new hypertension patients, or noting a history of taking diuretics, should always enquire about consumption or use of any liquorice containing product [14]. Health care workers should update their knowledge about which drugs their patients are using, and follow-up on unwanted or toxic side effects [40, 41].

## 6. Conclusion

Unduly stained teeth, a stained tongue or other oro-dental signs of intraoral chewing tobacco abuse combined with elevated blood pressures, should alert dentists to the possibility of morbidity arising from liquorice toxicity or abuse. 

With regular dental maintenance, regular medical history updates are essential and dental practices should measure blood pressure before any surgery. Some patients may even volunteer information about raised blood pressure. Health care professionals should check diets of all hypertensive patients and besides giving advice about eschewing liquorice products could refer affected people for early diagnosis and treatment for hypertension. 

## References

[1] Brown D (1995). *Encyclopaedia of Herbs and Their Uses*.

[2] Olukoga A, Donaldson D (2000). Liquorice and its health implications. *Journal of the Royal Society for the Promotion of Health*.

[3] Budavari S (1996). *Chemical Formulations. Glycyrrhizin-4515. Cyclamate-2770; Saccharin-8463*.

[4] Quinkler M, Stewart PM (2003). Hypertension and the cortisol-cortisone shuttle. *The Journal of Clinical Endocrinology & Metabolism*.

[5] Snow J (1996). *Glycyrrhiza glabra*. Monograph. *Protocol Journal of Botanical Medicine*.

[6] Acharya SK, Dasarathy S, Tandon A, Joshi YK, Tandon BN (1993). A preliminary open trial on interferon stimulator (SNMC) derived from *Glycyrrhiza glabra* in the treatment of subacute hepatic failure. *Indian Journal of Medical Research*.

[7] Arase Y, Ikeda K, Murashima N (1997). The long term efficacy of glycyrrhizin in chronic hepatitis C patients. *Cancer*.

[8] Van Rossum TGJ, Vulto AG, Hop WCJ, Brouwer JT, Niesters HGM, Schalm SW (1999). Intravenous glycyrrhizin for the treatment of chronic hepatitis C: a double-blind, randomized, placebo-controlled phase I/II trial. *Journal of Gastroenterology and Hepatology*.

[9] Van Rossum TGJ, Vulto AG, Hop WCJ, Schalm SW (2001). Glycyrrhizin-induced reduction of ALT in European patients with chronic hepatitis C. *American Journal of Gastroenterology*.

[10] Farese RV, Biglieri EG, Shackleton CHL, Irony I, Gomez-Fontes R (1991). Licorice-induced hypermineralocorticoidism. *The New England Journal of Medicine*.

[11] Rotblatt M, Zimenr I (2002). Liquorice. *Evidence Based Herbal Medicine*.

[13] Aoki F, Nakagawa K, Kitano M (2007). Clinical safety of Licorice Flavonoid Oil (LFO) and pharmacokinetics of glabridin in healthy humans. *Journal of the American College of Nutrition*.

[14] Sigurjónsdóttir HÁ, Franzson L, Manhem K, Ragnarsson J, Sigurdsson G, Wallerstedt S (2001). Liquorice-induced rise in blood pressure: a linear dose-response relationship. *Journal of Human Hypertension*.

[15] Kannel WB (2000). Fifty years of Framingham Study contributions to understanding hypertension. *Journal of Human Hypertension*.

[16] Adam L (1997). In vitro antiviral activity of indigenous glycyrrhizin, liquorice and glycyrrhizic acid (Sigma) on Japanese encephalitis virus. *Journal of Communication Disorders*.

[17] Cinatl J, Morgenstern B, Bauer G, Chandra P, Rabenau H, Doerr HW (2003). Glycyrrhizin, an active component of liquorice roots, and replication of SARS-associated coronavirus. *The Lancet*.

[18] Hattori T, Ikematsu S, Koito A (1989). Preliminary evidence for inhibitory effect of glycyrrhizin on HIV replication in patients with AIDS. *Antiviral Research*.

[19] Chung JG (1998). Inhibitory actions of glycyrrhizic acid on arylamine N-acetyl transferase of *Helicobacter pylori* from peptic ulcer patients. *Drug and Chemical Toxicology*.

[20] Rees WDW, Rhodes J, Wright JE (1979). Effect of deglycyrrhizinated liquorice on gastric mucosal damage by aspirin. *Scandinavian Journal of Gastroenterology*.

[21] Borrelli F, Izzo AA (2000). The plant kingdom as a source of anti-ulcer remedies. *Phytotherapy Research*.

[22] Shintani S, Murase H, Tsukagoshi H, Shiigai T (1992). Glycyrrhizin (licorice)-induced hypokalemic myopathy: report of 2 cases and review of the literature. *European Neurology*.

[23] Tyler VE (1994). *Herbs of Choice: The Therapeutic Use of Phytomedicinals*.

[24] Shibata S (2000). A drug over the millennia: pharmacognosy, chemistry, and pharmacology of licorice. *Yakugaku Zasshi*.

[25] Abdollahi M, Rahimi R, Radfar M (2008). Current opinion on drug-induced oral reactions: a comprehensive review. *Journal of Contemporary Dental Practice*.

[26] Atilla K, Vasan RS (2006). Prehypertension and risk of cardiovascular disease. *Expert Review of Cardiovascular Therapy*.

[27] Saydah S, Eberhardt M, Rios-Burrows N, Williams D, Geiss L, Dorsey R (2007). Prevalence of chronic kidney disease and associated risk factors—United States, 1999–2004. *Morbidity and Mortality Weekly Report*.

[28] Ong KL, Cheung BMY, Man YB, Lau CP, Lam KSL (2007). Prevalence, awareness, treatment, and control of hypertension among United States adults 1999–2004. *Hypertension*.

[29] Moore J (2005). Hypertension: catching the silent killer. *The Nurse Practitioner*.

[30] Mumoli N, Cei M (2008). Licorice-induced hypokalemia. *International Journal of Cardiology*.

[31] van Uum SH (2005). Liquorice and hypertension. *The Netherlands Journal of Medicine*.

[32] Russo S, Mastropasqua M, Mosetti MA, Persegani C, Paggi A (2000). Low doses of liquorice can induce hypertension encephalopathy. *American Journal of Nephrology*.

[33] Saito T, Tsuboi Y, Fujisawa G (1994). An autopsy case of licorice-induced hypokalemic rhabdomyolysis associated with acute renal failure: special reference to profound calcium deposition in skeletal and cardiac muscle. *Nippon Jinzo Gakkai Shi*.

[34] Breidthardt T, Namdar M, Hess B (2006). A hypertensive urgency induced by the continuous intake of a herbal remedy containing liquorice. *Journal of Human Hypertension*.

[35] de Klerk GJ, Nieuwenhuis MG, Beutler JJ (1997). Hypokalaemia and hypertension associated with use of liquorice flavoured chewing gum. *British Medical Journal*.

[36] Dluhy RG, Anderson B, Harlin B, Ingelfinger J, Lifton R (2001). Glucocorticoid-remediable aldosteronism is associated with severe hypertension in early childhood. *Journal of Pediatrics*.

[37] Lifton RP (2004). Genetic dissection of human blood pressure variation: common pathways from rare phenotypes. *Harvey Lectures*.

[38] Armanini D, Calò L, Semplicini A (2003). Pseudohyperaldosteronism: pathogenetic mechanisms. *Critical Reviews in Clinical Laboratory Sciences*.

[39] Sontia B, Mooney J, Gaudet L, Touyz RM (2008). Pseudohyperaldosteronism, liquorice, and hypertension. *The Journal of Clinical Hypertension*.

[40] Touyz LZG (2007). Liquorice, hypertension and the dentist. *The Journal of the Canadian Dental Association*.

[41] Vaya J, Belinky PA, Aviram M (1997). Antioxidant constituents from liquorice roots: structure elucidation and anti-oxidative capacity towards LDL oxidation. *Free Radical Biology & Medicine*.

